# Population-level Outcomes of Early Thyroid Cancers: A Need to Revisit Current Practice

**DOI:** 10.5041/RMMJ.10467

**Published:** 2022-04-26

**Authors:** Pankaj Chaturvedi, Arjun Singh, Atanu Bhattacharjee, Vidisha Tuljapurkar, Deepa Nair, Devendra Chaukar, Rajesh Dikshit

**Affiliations:** 1 Department of Head and Neck Oncology, Tata Memorial Centre and HBNI, Mumbai, India; 2 Section of Biostatistics, Centre for Cancer Epidemiology, Tata Memorial Centre and HBNI, Mumbai, India; 3 Centre for Cancer Epidemiology, Tata Memorial Centre and HBNI, Mumbai, India

**Keywords:** Early cancer, observation, SEER database, survival outcomes, thyroid cancer

## Abstract

**Background:**

Early thyroid cancers have excellent long-term outcomes, yet the word “cancer” draws unnecessary apprehension. This study aimed to define when the recommendations for observation and surveillance may be extended to early thyroid cancers at the population level.

**Methods:**

Non-metastasized thyroid cancers ≤40 mm diameter were identified from the 1975–2016 Surveillance, Epidemiology and End Results (SEER) database. Causes of death were compared across demographic data. Disease-specific outcomes were compared to the age-adjusted healthy United States (US) population. Survival estimates were computed using Kaplan–Meier and compared using the Cox proportional hazard model. Dynamic benchmarks impacting disease-specific overall survival were determined by decision tree modeling and tested by the Cox model.

**Results:**

Of the 28,728 thyroid cancers included in this study, 98.4% underwent some form of thyroid-specific treatment and were followed for a maximum of 10.9 years. This group had a 4.3% mortality rate at the end of follow-up (10.9 years maximum), with 13 times more deaths attributed to competing risks rather than thyroid cancer (stage T1a versus stage T1b, *P*=1.000; T1 versus T2, *P*<0.001). Among the untreated T1a or T1b tumors, the risk of disease-specific death was 21 times lower than death due to other causes. There was no significant difference between T1a and T1b tumors nor across sex. The age-adjusted risk of death for the healthy US population was higher than for the population with thyroid cancer. Dynamic categorization demonstrated worsening outcomes up to 73 years, uninfluenced by sex or tumor size. For patients over 73 years of age, only tumors >26 mm impacted outcomes.

**Conclusion:**

Based on the current data, T1a and T1b nodules have similar survival outcomes and are not significantly impacted even when left untreated. Multi-institutional prospective studies are needed to confirm these findings so that current observation and surveillance recommendations can be extended to certain T1 thyroid nodules.

## INTRODUCTION

Thyroid cancer is the most common endocrine malignancy today, with an incidence of above half a million cases worldwide.^1^ Since a 5-year relative survival rate of 100% has been reported for these patients, people with thyroid cancers and those who are more likely to be screened for the disease might be considered healthier, with a lower risk of dying than the general population.^2^ Despite detailed treatment guidelines, controversy continues to revolve around the definition and management of “early” thyroid cancer, which might not need surgery.^3^ This early disease is often incidentally detected, indolent, and diagnosed at a younger age than most other adult cancers.^3^,^4^ Even when thyroid cancer is left untreated after diagnosis, survival is similar to those not receiving thyroid-specific cancer treatment.^5^–^7^

The incidence of thyroid cancer has nearly tripled in the United States, primarily owing to over-screening and incidental detection.^8^ Theoretically, this screen-and-treat strategy should result in early detection and treatment of low-volume nodules, eventually translating into better survival outcomes. However, this has been proved otherwise, with studies reporting no cost-benefit for low-volume carcinomas due to excellent long-term survival even when left untreated.^5^,^9^ Moreover, the population-level mortality due to thyroid cancer has remained quite low and unchanged over the last several decades, despite major disease-related advances, drawing criticism for their unwarranted screening.^10^

The staging of differentiated thyroid cancer is unique among adult malignancies as it incorporates an individual’s age.^11^ Recently, the prognosticative age was raised to 55 years for all thyroid cancers as it led to the downstaging of a significant percentage of patients from the previously used benchmark of 45 years.^12^ Currently, only patients over 55 years are conventionally staged according to tumor size and metastatic patterns, similar to other head and neck sites. The impact of sex on incidence, biology, and outcomes has also been extensively reported for thyroid cancer. Thyroid cancer is almost three times more common in women and is believed to have better outcomes in women, probably due to aggressive histology and advanced stage presenting more commonly in males.^13^ A decade ago, the American Joint Committee on Cancer (AJCC) divided stage T1 (≤2 cm) into two groups: T1a (≤1 cm; microcarcinomas) and T1b (>1 cm but ≤2 cm).^11^ Today, the latest American Thyroid Association (ATA) guidelines recommend diagnostic intervention for only those nodules >1 cm, i.e. ≥T1b.^3^ Interestingly, small case studies also suggest no significant decline in outcomes until the tumor reached stage T2 (>40 mm), irrespective of management type.^14^,^15^ Based on such evidence, certain thyroid “cancer” variants have been reclassified into “neoplasms,” such as the non-invasive follicular thyroid neoplasm with papillary-like nuclear features, possibly to avoid unnecessary apprehension.

The controversy does not end with investigating early thyroid cancers but extends into management, where the extent of surgery is still debated. Considering the slow growth and extremely low mortality, a more appropriate definition is needed for “micro” or early thyroid cancer to avoid unwarranted investigations and treatment. This study aimed to determine such a definition by incorporating the collective impact of tumor size, age, and sex on survival outcomes and investigate the possibility of extending the recommendation for observation and surveillance to the rest of the low-volume thyroid cancers.

## METHODS

The 1975–2016 Surveillance, Epidemiology and End Results (SEER) database was used to obtain demographic, disease, and survival outcome information.^16^ Population-based data were used for these analyses since it provided a true reflection of real-world disease outcomes as opposed to hospital-based data that are likely to have inherent biases. Patients with thyroid cancer were identified according to the topography and histology codes of the International Classification of Diseases for Oncology, 3rd ed. (ICD-O-3).^17^ The pathologic tumor size was considered for subjects who had undergone thyroid-specific treatment. For subjects who did not receive thyroid-specific treatment, the clinical tumor size was considered according to the SEER staging database. Specific investigations performed to determine the size and staging were not mentioned in the database. Subjects with a tumor size ≤40 mm were included. Individuals were excluded if there was metastasis to regional or distant sites or if metastatic status was not mentioned. The study group was then stratified according to the AJCC classifications, i.e. stages T1a, T1b, and T2. Since a population-level analysis was planned, the full spectrum of thyroid cancer histologies was considered.

The SEER database uses algorithms to process the cause of death from death certificates in order to identify a single underlying cause of death and is highly reliable. It presents two variables for estimating cause-specific survival probability due to “cancer” or “other causes.” Therefore, disease-specific survival (DSS) was reliably defined as the time from diagnosis to the time of death due to thyroid cancer or last follow-up, whichever occurred first. The authors stratified all cancer-specific causes into two categories: thyroid cancer-specific and competing risks (i.e. all others, including other cancers). These outcomes were compared across age and gender against the causes of death. Survival comparison was made between the age-adjusted US population, obtained from the National Center for Health Statistics and the Center for Disease Control (CDC), and the thyroid-specific deaths obtained from the SEER registries.^18^ Tree modeling using R-software (https://cran.r-project.org/) determined dynamic benchmarks for age, gender, and tumor size, based on their weightage as predictors of death. Estimates and confidence intervals (CI) of overall survival proportions were obtained using the Kaplan–Meier method, and survival distributions were compared across groups using the log-rank test. Cox proportional hazards regression analysis was used to evaluate the risk of death, i.e. hazard rate (HR), across tumor size, age, and sex. A two-sided *P*-value of <0.05 indicated statistical significance.

## RESULTS

**Figure 1:**
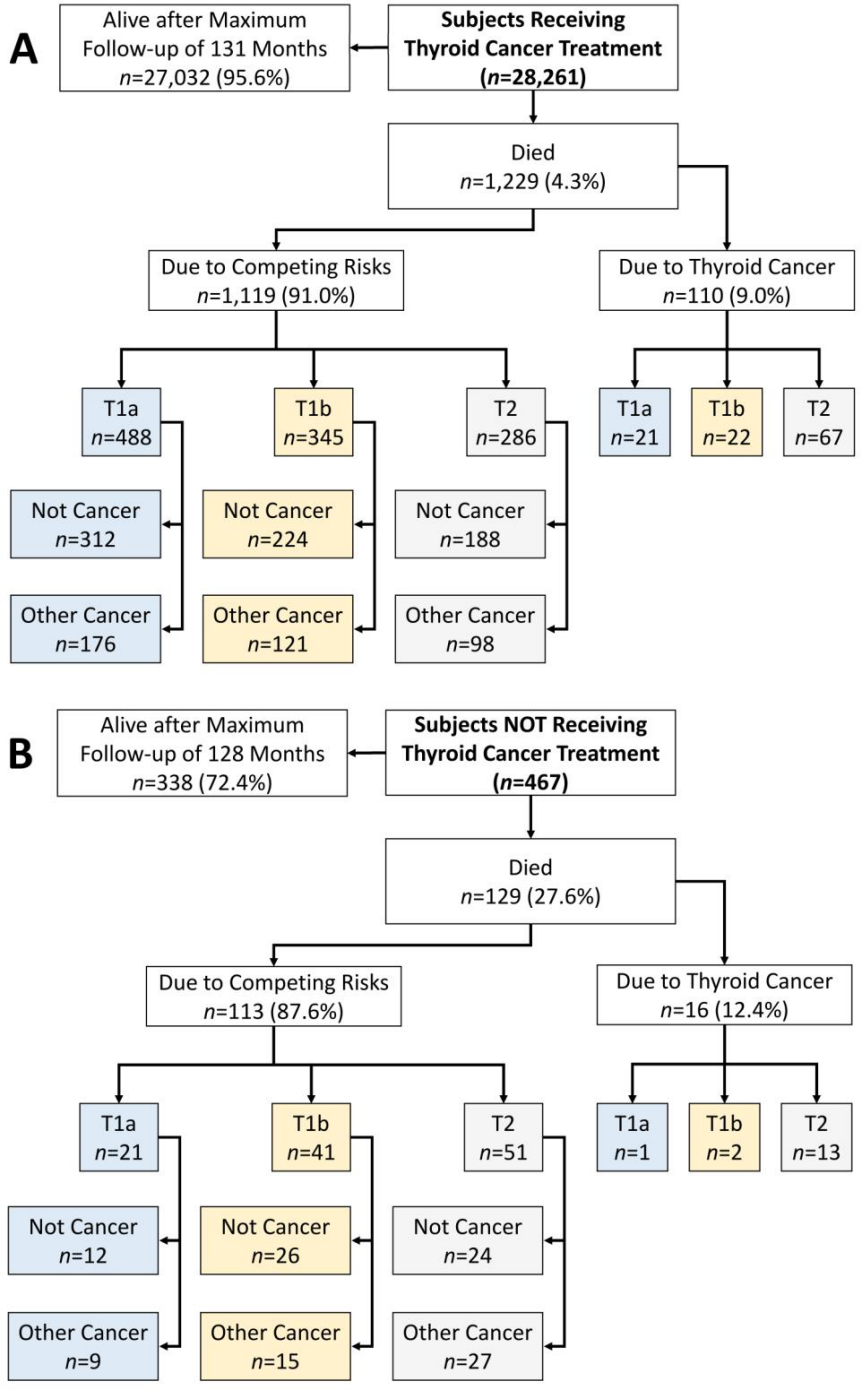
Overview of Subjects in the SEER Database (Tumor Size ≤40 mm, Node Absent, Metastases Absent) with Distribution of Death According to Tumor Size and Treatment. **A:** Subjects who received thyroid cancer-directed treatment. **B:** Subjects who did not receive thyroid cancer-directed treatment. T1a, tumor size ≤10 mm; T1b, tumor size >10 mm to ≤20 mm; T2, tumor size >20 mm to ≤40 mm.

Of the 43,212 thyroid cancers registered in the 1975–2016 SEER database, 28,728 cases fulfilled the inclusion criteria of tumor size ≤40 mm with no local (lymph node, N0) or distant (M0) metastasis, i.e. T1aN0M0, T1bN0M0, and T2N0M0 (Table 1 and Figure 1). Of these, 98.4% patients (*n*=28,261) underwent some form of thyroid or disease-specific treatment and 1.6% (*n*=467) did not. Of those undergoing some form of treatment, 47.9% (13,529/28,261) were staged T1a, 29.6% (*n*=8369/28,261) were staged T1b, and 22.5% (*n*=6363/28,261) were staged T2 tumors.

**Table 1:** Clinical-demographic Comparison of the Treated (*n*=28,261) versus Non-treated (*n*=467) Groups.

Parameter	Treated Group (*n*=28,261)	Non-treated Group (*n*=467)	*P* Value
Mean follow-up in months (range)	55.70 (0–131)	35.23 (0–128)	<0.001

Mean age in years (range)			
at diagnosis	50.1 (0–105)	57.7 (20–95)	<0.001
at death	68.8 (18.7–101.2)	71.2 (40.7–95.5)	0.06
at end of follow-up*	54.1 (4.5–105)	56.7 (20–96)	0.001

Sex			
Male	5,489	143	<0.001
Female	22,772	324	

Cause of death			
Thyroid cancer	110	16	<0.001
Other	1,119	113	<0.001

* This group includes only those subjects living at the end of follow-up for the SEER study and therefore excludes those who died from any cause during follow-up.

Of the 28,261 cases that received disease-specific treatment regardless of histology and treatment type, 95.6% survived after a maximum follow-up of 10.9 years (median, 4.3 years; mean, 4.6 years). The mean age of this group was 50.1 years. The total deaths amongst those who received treatment was 4.3%) (*n*=1,229/28,261). The cause of death was thyroid specific in 0.4% (*n*=110/28,261) and due to competing interests in 4.0% (1,119/28,261). Among the disease-specific deaths, only 3.5% were T1 tumors. Although no difference was noted between T1a and T1b for disease-specific deaths (T1a versus T1b; *P*=1.000), there was a significant difference when comparing the total numbers of T1 (T1a, 0.07%; and T1b, 0.08%) and T2 (0.24%) tumors (T1 versus T2, *P*<0.001). There was a 10 times higher risk of death for the group receiving thyroid-specific cancer treatment due to competing causes. That risk became 23 times higher when only T1a tumors were considered, while for T1b the risk was 15 times higher than the risk of death in the sub-group that died due to thyroid cancer alone.

Of the patients who received no disease-specific treatment (*n*=467), 27.6% (*n*=129) had died by the end of a maximum follow-up of 10.6 years (median=2.1 years, mean=2.9 years). The group’s mean age was 57.7 years, which was not significantly different from those who received thyroid-specific treatment. Only 3.4% (*n*=16) died due to thyroid-specific causes. These deaths comprised a single T1a tumor in an 88-year-old woman, two T1b tumors (mean patient age 66.5 years), and 13 T2 tumors (mean patient age 74.7 years). The remaining 87.5% (*n*=113) died due to competing risks. The risk of dying due to thyroid cancer in a person with an untreated T1a or T1b tumor was approximately 21 times lower than dying due to other causes.

**Figure 2:**
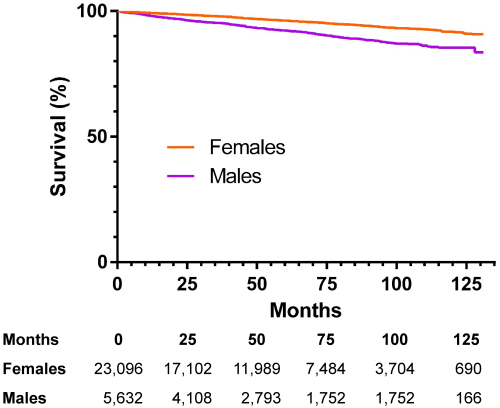
Thyroid-specific Survival Outcomes Based on Sex Among Subjects Who Received Some Form of Thyroid Cancer-directed Treatment.

**Figure 3:**
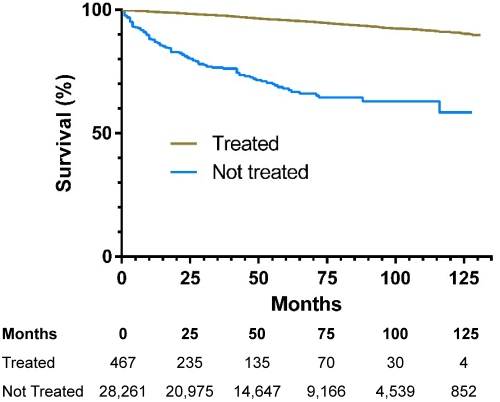
Thyroid-specific Survival Outcomes and Kaplan-Meier Curves for the Treated and Not Treated Subjects.

Analyzing outcomes for each millimeter of tumor progression, the risk of thyroid-specific death significantly increased only after the tumor size exceeded 20 mm in the group that did not receive disease-specific treatment and above 30 mm for the group that did receive disease-specific treatment. Considering the conventional AJCC classification, there was no significant difference in risk of disease-specific death between T1a and T1b in both the treated and untreated groups (Tables 2 and 3). Males had a significantly larger proportion of death due to disease-specific (0.58% versus 0.34% in females, *P*<0.001) and competing causes (6.7% versus 3.3% in females, *P*<0.001) (Figure 2). A significant difference between treated and untreated groups was observed (Figure 3). However, as tumor size increased, there was no significant difference between sexes, even though the absolute number of females was higher (*P*=0.62). Comparing these outcomes to the age-adjusted healthy US population, the risk of death for the general population was higher than for the population having thyroid cancer for both sexes.

**Table 2:** Difference in Thyroid Cancer-specific Deaths Between the Treated (*n*=28,261) and Non-treated (*n*=467) T1a and T1b Groups.

Tumor Size(Total Subjects)	Thyroid Cancer-specific Deaths		*P* Value*
	Treated Group	Non-Treated Group	
T1a (≤10 mm) (*n*=13,634)	0.16% (21/13,529)	0.95% (1/105)	0.16
T1b (>10 to ≤20 mm) (*n*=5,558)	0.41% (22/5,369)	1.06% (2/189)	1.0

* Calculated based on time-to-event.

**Table 3:** Cox Analysis Hazard Rate (HR) Estimates for Progressing Tumor Size.

Tumor Size	Received Some Form of Thyroid Cancer-directed Treatment (*n*=28,261)			Did Not Receive Any Thyroid Cancer-directed Treatment (*n*=467)		
	HR (adjusted)	*P* Value	95% CI	HR (adjusted)	*P* Value	95% CI
T1a (≤10 mm)	Ref	-	-	Ref	-	-
T1b (>10 to ≤20 mm)	0.926	0.77	(0.55, 1.54)	1.12	0.08	(0.98, 1.28)
T2 (>20 to ≤30 mm)*	1.393	0.21	(0.82, 2.35)	1.23	**0.01**	(1.04, 1.44)
T2 (>30 to ≤40 mm)*	1.784	**0.04**	(1.01, 3.14)	1.71	0.00	(1.43, 2.06)

* For purposes of analysis the T2 group was split into two groups based on size.

The collective impact of age, sex, and tumor size (0–40 mm) on DSS was determined using a real-world data-driven categorization rather than dividing the groups with the conventional AJCC grouping. In this decision tree, the thickness of the stem demonstrated the predicted response value. Only patients that received thyroid-specific treatments were included for this analysis. Age was the most influential factor, followed by tumor size. The best outcomes were seen in the patients under 15 years of age (*n*=103) with progressive worsening up to 73 years, irrespective of sex or tumor size. In the group above 73 years of age, tumor size >26 mm played a significant role regardless of sex. A stronger disease-specific hazard discrimination was observed for the data-driven cut-off value grouping of 0–26 mm and 27–40 mm (HR, 1.516; 95% CI 1.315, 1.748; *P*<0.001) than the conventional T1 and T2 classifications, i.e. 0–20 mm and 20–40 mm (HR, 1.330; 95% CI 1.175, 1.505; *P*<0.001). A similar method was applied that considered only tumors in the range 0–20 mm, i.e. T1a and T1b. Here, age was the most influential factor with a minimal significance of tumor size. The best outcomes were seen in individuals below the age of 86 years. In patients above this age, tumor size >18 mm contributed toward a worse DSS.

## DISCUSSION

Malignancy is a term used for tumors that uncontrollably divide and metastasize to distant tissues of the body in a relatively short amount of time.^19^,^20^ On the other hand, benign tumors lack this ability to metastasize, staying dormant and rarely growing at a noticeable pace.^19^,^20^ Most thyroid cancers are traditionally known to have an intermediary behavior.^4^ They encompass several histologic variants that occur due to transformation of the follicular and C-cells and various mutations that encode different effector signals.^21^,^22^ Although some harbor the potential to be aggressive, most start unnoticed and often remain indolent.^3^,^23^ The primary complaint is often of a prominent lump in the neck gradually increasing in size over several months or years. Early on, they rarely become clinically significant since the majority are differentiated cancers with fewer copy-number alterations.^21^ Using whole-exome sequencing, some variants have been shown to have one of the lowest mutation densities among all cancers, making them clinically dormant for long periods.^24^

In the past few decades, the incidence of thyroid cancer has significantly increased, with mortality remaining negligible. These cancers are popularly over-diagnosed, largely due to the unnecessary detection of subclinical disease by sensitive diagnostic procedures. Incidentalomas are detected on unrelated investigations such as breast ultrasounds, carotid Doppler studies, and head and neck CT, MRI, and PET scans. Since these nodules are not otherwise detected, if not symptomatic or picked up on screening, their prevalence in the general population comes only from autopsy studies, ranging from 2% to 36%.^25^,^26^ In general, about one-third of the adult population may harbor indolent, sub-centimeter papillary thyroid carcinomas.^21^

Microcarcinoma is a term used commonly for T1a size tumors.^27^ Large database analyses have demonstrated 99.9% 15-year DSS for papillary microcarcinomas.^28^ Another study of more than 2,000 patients has reported a mortality rate of 0.6% among papillary microcarcinomas during a follow-up period of 16.5 years.^29^ Even compared to the age-matched normative US population, these cancers demonstrate comparable survival outcomes regardless of the treatment strategy undertaken.^30^ Based on similar reports, most international guidelines suggest observing nodules less than 10 mm without any active diagnostic intervention. In general, all variants of early thyroid cancer begin with a similar clinical presentation, and it is almost impossible to differentiate histology based on clinical findings alone at this stage. Hence, our population-level analysis included all thyroid cancer variants, including the aggressive histology. Among T1a tumors, we found the risk of death attributable to thyroid cancer to be only 74 per 100,000 early thyroid cancers—almost 23 times lower than the risk of death due to other causes (1,727 per 100,000 early thyroid cancers). These findings reiterate that T1a tumors have an extremely low disease-specific mortality. Hence, it is justified to continue observing certain T1a nodules.

Irrespective of histology and the type of treatment received, or no treatment, our results demonstrate no statistically significant difference in the DSS between T1a and T1b tumors. When tumors ranging from 0 to 20 mm were considered in the decision tree modeling, the difference was seen at a very advanced age of more than 86 years, which was for a tumor size greater than 18 mm. These findings suggest that T1a and T1b tumors might behave similarly and can be considered a single entity, extending the possibility for observation to T1b tumors. Interestingly, there was a significant difference in the DSS when the tumor size increased from T1 (0–20 mm) to T2 (20–40 mm). The data-driven classification suggests that similar tumor behavior is seen up to 26 mm, with a stronger discriminative hazard difference using this cut-off. Since outcomes worsen among those over 73 years with tumors larger than 26 mm, the observation criteria can be extended further—up to 26 mm. To validate our findings of similar outcomes among T1a and T1b nodules, we analyzed the 467 patients who had not received any disease-specific treatment. This tumor subgroup represents the natural course of the disease over time. The DSS among these cases remained relatively unchanged as the tumor size increased from T1a to T1b. Similar findings have been reported in another study of 2,638 patients with clinical T1N0M0 papillary carcinomas; the authors found no difference in 10-year disease-free survival between T1a and T1b cancers (97% for both groups).^14^ Two large Japanese studies also strongly suggest the benign nature of thyroid microcarcinomas and the safety of active surveillance in these cases.^6^,^31^ Both studies have shown that after 5—10 years of observation, only 7%–8% of the tumors grew by 3 mm or more, and only 1%–3.8% had developed nodal metastasis. Even among the patients who had surgery after a period of observation because of tumor growth, development of lateral nodes, or patient preference, not one patient had died or developed distant metastases, and only one had a recurrence in a thyroid remnant.^6^ A meta-analysis of 17 studies has compared 854 incidental and 2,669 non-incidental micropapillary cancers with an average follow-up of 70 months.^32^ Overall recurrence rate was 7.9% in the non-incidental group and 0.45% in the incidental group. The reported mortality of 0.1% in the non-incidental group, with no deaths reported in the incidental group, along with the results mentioned in our study, shows that even if T1a cancers are left untreated, the risk of death due to the disease itself is negligible.

Ours is the first population-level study that has demonstrated that lesions between 10 mm to 20 mm (T1b) have outcomes similar to T1a tumors, irrespective of any form of treatment received or no treatment. It is important to interpret these results with caution and only as a hypothesis-generating possibility of active surveillance for certain T1b tumors; level 1 evidence from multicenter prospective data is needed to corroborate these findings. Although the current study did not intend to analyze and report the outcomes of the pediatric population, a significant survival advantage was seen in patients below 15 years of age. This is probably due to including a few outlying tumors that were detected and received treatment at an unusually early stage rather than the conventional presentation of pediatric thyroid cancer.

Since T1 thyroid cancers have an excellent DSS, their designation as a malignancy significantly impacts the quality of life. Even when these outcomes were compared against the age-adjusted healthy US population, the risk of death for the general population was higher than for those with thyroid cancer among both sexes. Furthermore, thyroid cancer has been shown to be associated with the highest bankruptcy rates among all cancers.^33^ Highlighting the above findings, it might be necessary to revisit the guidelines in the future to extend observation and possibly surveillance to all T1 thyroid cancers, rather than T1a alone. Based on the database used for our study, there was a beneficial effect of intervention only for those individuals above 73 years and with nodules larger than 26 mm, which effectively extends the current guidelines.^3^ The data serve as preliminary support for a possible change in the management of early thyroid cancers. The limitations of our study include: SEER database entry errors, lack of information on the thyroid-specific treatment received that would have impacted survival, inclusion or exclusion of cases that moved from or received treatment outside of the SEER regions, unknown reasons for patients not receiving treatment, and possible misattribution of a single cause of death that may be difficult in some situations.

## CONCLUSION

The current study provides evidence for similar survival outcomes of T1a, T1b, and certain T2 tumors defined by the current AJCC classification, i.e. they are not significantly impacted even when left untreated. Multi-institutional prospective studies are needed to confirm these findings so that the current recommendations of observation and surveillance can be extended to other early thyroid nodules.

## Abbreviations

AJCC: American Joint Committee on Cancer
ATA: American Thyroid Association
CI: confidence interval(s)
DSS: disease-specific survival
HR: hazard rate
ICD-O-3: International Classification of Diseases for Oncology
SEER: Surveillance, Epidemiology and End Results
US: United States
